# Editorial: Computational Methods for Microbiome Analysis

**DOI:** 10.3389/fgene.2020.623897

**Published:** 2020-12-11

**Authors:** João C. Setubal, Jens Stoye, Bas E. Dutilh

**Affiliations:** ^1^Department of Biochemistry, Institute of Chemistry, University of São Paulo, São Paulo, Brazil; ^2^Faculty of Technology and Center for Biotechnology (CeBiTec), Bielefeld University, Bielefeld, Germany; ^3^Theoretical Biology and Bioinformatics, Department of Biology, Science for Life, Utrecht University, Utrecht, Netherlands

**Keywords:** bioinformatics, metagenomics, microbiome, microbial ecology, taxonomic classification

Microbes play critical roles in the lives of hosts (plants, animals, humans) and in almost any environment one can think of. Gathering microbiome sequence data has become easier and cheaper than ever before, leading to an exponential growth in the amount of such data available for analysis. With this explosion has come a pressing need for sophisticated computational tools that can help make sense of these datasets. Current challenges, such as the complexity of microbiome-host-environment interactions and the large sizes of datasets, make for a fascinating research field.

The goal of this Research Topic was to gather a collection of high-quality original papers on the general theme of computational methods for microbiome analysis. We now present the results, which consist of 13 papers.

Four papers consider amplicon analysis, a popular method for taxonomic classification of mixed microbial samples based e.g., on 16S rRNA gene regions. The paper by Engelmann et al. describes Cascabel, a software pipeline for automated processing and analyzing of massive amounts of amplicon data. Cascabel wraps around existing and well-established tools in the field of amplicon analysis, connecting them by means of a Snakemake workflow, thus allowing for an easy and flexible execution of a common amplicon analysis pipeline. After a workflow is finished, reports are generated also serving as a data provenance description.

Similar in flavor is NG-Tax 2.0, described in the paper by Poncheewin et al. which follows the new amplicon sequencing variant (ASV) approach, where sequencing reads are grouped into ASV clusters of very high similarity in order to sustain as much as possible the true biological variance in the sample at hand. NG-Tax 2.0 performs several steps in a pipeline manner, which includes demultiplexing, read cleaning, ASV clustering, and taxonomic classification.

In a somewhat theoretical study based on computer simulations, Pinna et al. try to answer the question of whether non-contiguous V-regions with paired-end sequencing improve 16S rRNA based taxonomic resolution of microbiomes. They explore the possibility of combining two regions of the 16S rRNA gene for a better classification of the tags and to possibly iron out the weakness of taxonomic resolution of one region by a higher resolved other region. And indeed, the combination of two distant variable regions shows on average 10-20 percent higher accuracy in taxonomic classification—a theoretical potential, however, that still needs to be explored in practice.

Still on the topic of taxonomic classification, Shah et al. present ATLAS, a novel strategy for taxonomic annotation of 16S rRNA sequence data. It has been recognized that 16S amplicon data does not in general allow reliable classification below the genus level. However, 16S sequence data and the accumulated knowledge on the diversity of this marker gene (as present in various 16S databases) may allow reliable classification at the “sub-genus level,” meaning classification that would suggest possible species, to the exclusion of others, of the same genus. That is the main achievement of the ATLAS pipeline, which is therefore a contribution for better use of the large amounts of 16S data already available and yet to come.

Moving on to other subtopics, Dong and Strous present MetaErg, a user-friendly platform to explore the information in complex metagenomic datasets. It facilitates the annotation, visualization, and interpretation of assembled and/or binned shotgun metagenomes by using taxonomic and functional annotation of genes identified in metagenomic contigs or metagenome-assembled genomes, or MAGs. Homology searches may be performed with DIAMOND-blast as well as a collection of HMM-profiles. Moreover, MetaErg allows the incorporation of additional -omics data such as metaproteomics to identify gene expression. The HTML-based output pages allow users to navigate through annotation tables, trees, and sunburst plots to explore their data.

Accurate metagenome assembly and genome binning from short-read data may be confounded by e.g., repeated regions and mobile genetic elements. Chromosomal contact data from meta3C or Hi-C experiments is a promising way to address these challenges. Baudry et al. present MetaTOR, a binning pipeline that uses contact frequencies to reconstruct MAGs from meta3C metagenomic libraries. Application to murine gut metagenomes enabled the recovery of MAGs corresponding to nearly a third of the total assembly data of 20 meta3C libraries, underlining the promise of chromosomal contact data for metagenome-binning and the potential to describe microbial communities with MAGs.

Hester et al. present a new metric for evaluating functional redundancy in metagenomes that they call metabolic overlap (MO). The metric needs annotated MAGs of each environment considered. They observed highest values of MO for aquatic and low pH/high temperature environments, and lowest values in communities associated with animal hosts, in one built/engineered environment, and in soil. It is an excellent example of an analysis method that seeks to unlock the rich information contained in MAGs to help understand competition and cooperation between species

Within the rapidly expanding field of microbiome science, it is becoming difficult to stay up-to-date with the literature on microbe-human interactions, as a lot of new information is being published. Srivastava et al. present EviMass, a new tool to gain information about microbial associations to the human superorganism from literature. Evimass consists of an interactive query system on top of a large database derived from mined microbe-microbe and disease-microbe associations from PubMed abstracts. Thus, by uploading their own microbial interaction data, users can link these associations to information from biomedical literature. Various output formats and statistics are available, allowing researchers to place their microbiome experiments among the wealth of information in the literature.

Gene-targeted assembly is a useful approach to identify and track specific genes in metagenomic datasets. Guo et al. present a benchmark comparing the computational efficiency, sensitivity, specificity, and chimera rate of six existing gene-targeted assembly tools. The authors focused on extracting the universal ribosomal protein rplB and two nitrogen cycle genes, dinitrogenase reductase gene nifH, and nitrite reductase gene nirK from testing datasets consisting of known genomes, synthetic and mock communities, and a large soil shotgun metagenome. They assessed assembly quality as well as computational performance of the tools. Two tools that employ probabilistic graph structures showed the best overall performance.

Metagenomics is providing unprecedented insights into our microbial world. Combined datasets generated by research laboratories around the world are opening up new opportunities to study the macroecological patterns on local-to-global scales. Mascarenhas et al. contributed a valuable and extensive review on the computational methods to investigate the macroecology of microbiomes. They address fundamental aspects of biodiversity, describe macroecological studies in the microbiology field, and stress how spatial and temporal sampling scales should fit the research question of each study. Next they describe methods including taxonomic profiling and co-occurrence networks, identifying keystones, and description of functional patterns. An important part of their review is a discussion of different approaches for predictive modeling which promise new insights in a range of fields.

To investigate the transcriptional activity of the microbial community, metatranscriptomics requires a specific subset of analysis tools. Shakya et al. review computational tools and recent advances in metatranscriptome analysis. Discussing metatranscriptomics studies investigating diverse ecosystems, they highlight the ability of metatranscriptomics to reveal the transcriptional activity of microbial communities with sometimes high resolution. The authors next discuss different bioinformatics tools and workflows including preprocessing, assembly, taxonomic and functional annotation, and differential expression analysis. They envision that the described tools will aid in the analysis of e.g., time series data to reveal the response of microbial communities to perturbations, although benchmarking is still needed.

Much emphasis has been given in the past to “snapshot” analysis of microbial communities, i.e., analysis of a community at a given point in time. However, for many environments, time is a crucial variable for the understanding of its microbial ecology. Hence, time-series sampling becomes an important strategy. This approach requires specialized techniques, which are nicely presented in the primer by Coenen et al. The authors describe several modules (interactive tutorials in R and Matlab) that address several topics in time-series analysis.

Van den Bogert et al. discuss various challenges for bioinformatics and data science in industrial microbiome applications. They review current applications and products in food, cosmetics and health industries. Some of the challenges facing these applications are also mentioned. Recent technological developments in the microbiome field are discussed and suggestions are given for how these developments could be leveraged to address certain challenges.

In sum, we believe the papers presented form a valuable collection for students and researchers working on the exciting and rapidly-growing field of microbiome analysis.

## Author Contributions

All authors listed have made a substantial, direct and intellectual contribution to the work, and approved it for publication.

## Conflict of Interest

The authors declare that the research was conducted in the absence of any commercial or financial relationships that could be construed as a potential conflict of interest.

